# The effect of counseling on fathers’ stress and anxiety during pregnancy: a randomized controlled clinical trial

**DOI:** 10.1186/s12888-021-03217-y

**Published:** 2021-04-23

**Authors:** Maryam Mohammadpour, Sakineh Mohammad-Alizadeh Charandabi, Jamileh Malakouti, Mehriar Nadar Mohammadi, Mojgan Mirghafourvand

**Affiliations:** 1 Midwifery Department, Tabriz University of Medical Sciences, Tabriz, Iran; 2 Faculty of Nursing and Midwifery, Tabriz University of Medical Sciences, Tabriz, Iran; 3 Faculty of Medicine, Ardabil University of Medical Sciences, Ardabil, Iran; 4 Social Determinants of Health Research Center, Faculty of Nursing and Midwifery, Tabriz University of Medical Sciences, Tabriz, Iran

**Keywords:** Anxiety, Stress, Counseling, Pregnancy

## Abstract

**Background:**

Pregnancy is a challenging period for mothers and fathers. This study aimed to investigate the effect of counseling on stress and anxiety levels of fathers.

**Methods:**

This randomized controlled trial was conducted on 102 spouses of pregnant women in Ardabil, Iran. The participants were randomly assigned to intervention and control groups. The intervention group attended four 60-min counseling sessions at weekly intervals. The perceived stress and anxiety questionnaires were completed before and 4 weeks after the intervention.

**Results:**

The mean scores of state anxiety in the intervention group decreased significantly 4 weeks after the intervention compared with the control group (MD: -2.4; 95%CI: − 4.7 to − 0.2; *p* = 0.030). Four weeks after the intervention, no significant difference was found between the two groups in terms of trait anxiety (*p* = 0.472) and perceived stress (*p* = 0.635).

**Conclusions:**

The findings indicate that counseling reduced state anxiety in expectant fathers; therefore, this intervention is recommended to be used to reduce fathers’ anxiety.

**Trial registration:**

IRCT2017042910324N38. Registered 25 June 2017

## Background

The transition to parenthood causes major psychological and social changes in future parents [1]. These changes are associated with increased anxiety, which is a common physiological problem among couples during pregnancy [2, 3]. Anxiety is an unpleasant and unknown mental state that emerges in the form of disorder, discomfort, excitement, and fear, and is accompanied by symptoms such as fatigue, restlessness, and palpitations [4].

Becoming a father is a major milestone in a men’s life and is described as a happy and promising moment [5]. Although fatherhood is a wonderful experience, fathers may sometimes experience unpleasant feelings [6]. In fact, men begin to communicate with their child during pregnancy, and this interaction creates a sense of reality, hope, and joy in fathers [5]. On the other hand, the position of a man in a family changes during pregnancy and after childbirth [4, 7]. Other factors, such as concerns about covering routine family expenses, new expenses, and concerns about reduced spouse income may also exacerbate the problem [8, 9]. In fact, men, like women, display signs of anxiety (although to a lesser extent) during pregnancy [10], and first-time expectant fathers experience higher levels of anxiety compared with others [11, 12]. Anxiety, irritability, and unrealistic emotions have been reported in men during the first months of pregnancy [8, 9].

Pregnancy is the most stressful time for fathers who experience high levels of anxiety during perinatal period [3, 13]. Stress refers to one’s inability to deal with his/her environment [14]. For some fathers, these stresses begin right after receiving the pregnancy test result; however, others may experience these feelings after hugging their baby for the first time [5]. Experimental studies report high levels of anxiety symptoms in fathers during pregnancy [3, 15, 16], and it seems that anxiety levels in men are higher during pregnancy and childbirth than in postpartum [1, 7]. As childbirth approaches, fear and concerns of men about the health of their wives and babies increase [17]. The results of a study showed that psychological distress is higher in men during pregnancy. This study also found that psychological distress is related to psychological variables, marital relationships, and poor support [14].

Although fathers enjoy getting involved in the childbirth process and attempt to actively support their wives, they also need support from healthcare professionals [18]; however, prenatal clinics mostly focus on maternal and infant health, and often ignore the needs and feelings of expectant fathers [6, 19]. At the beginning of the pregnancy period, fathers seem to need special counseling, particularly from experienced professionals [9]. Healthcare providers can better meet the needs of men during pregnancy and childbirth by increasing their knowledge and understanding men’s expectations and experiences during this period [14].

Considering that anxiety and stress are common psychological disorders among men during pregnancy and childbirth [20], which can affect the health of mother, fetus, and future baby [12], in addition, paying more attention to emotional problems of fathers effectively prevents their mental and emotional problems during pregnancy and postpartum [21] and since the researchers found no relevant study; therefore, the present research aimed to investigate the effect of counseling on stress and anxiety of expectant fathers.

## Methods

### Study type and participants

This randomized controlled trial with two parallel arms was performed on 102 spouses of pregnant women admitted to the health care centers of Ardabil, Iran, between July 2017 and July 2018.

Women with a gestational age of 20–24 weeks and singleton first pregnancy whose husbands were willing to attend counseling sessions and had not experienced a disastrous life event in the past 3 months and obtained a poor and moderate perceived social support score (125 or less) were included. The exclusion criteria included history of mental illnesses, taking psychiatric or psychotropic drugs, history of depression, obstetric problem (e.g. the possibility of a preterm delivery, diabetes, hypertension), and having an unwanted pregnancy.

This study is part of a larger study which investigates the effect of counseling to expectant fathers on social support, perceived stress, anxiety, and depression in pregnant women as primary outcomes and its effect on perceived anxiety and stress in men as secondary outcomes. The sample size was calculated according to the study of Iranzad et al. [22], based on both variables, namely perceived social support and perceived stress. First, based on the perceived stress variable and considering m1 = 11.5, the sample size was calculated 46 with the presumption of 25% decrease in the perceived stress score due to intervention (m2 = 8.6), sd1 = sd2 = 5.5, α = 0.05, power = 80%; then based on the social support variable and considering m1 = 69.6, the sample size was calculated 16 with the presumption of 25% increase in the social support score due to intervention (m2 = 52.2), sd1 = sd2 = 5.5, α = 0.05, power = 95%. Since the sample size calculated based on the perceived stress variable was higher, the final sample size was considered to be 51 in each group considering 10% attrition.

### Sampling and random assignment

This trial was conducted on spouses of primiparous pregnant women of childbearing age (15–49 years-old) visiting health centers and bases of Ardabil. Sampling was performed after obtaining permission from the Ethics Committee of Tabriz University of Medical Sciences (Ethics code: IR.TBZMED.REC.1396.24), registration of the study at the Iranian Registry of Clinical Trials (code: IRCT2017042910324N38) and coordination and obtaining permission from Ardabil health center.

The researcher attended the centers and bases and selected pregnant women with gestational age of 20 to 24 weeks according to their health records. To exclude abortion cases and occurrence of most physical and mental changes after the 20th week of pregnancy [23], 20 to 24 weeks were selected for sampling.

After examining the inclusion and exclusion criteria through a phone call and a brief explanation of the research and its importance, they were invited to visit the health center or base with their spouses on the specified date. After the presence of pregnant women and their spouses, the researcher introduced herself and explained the general objectives and stages of the study and examined the eligibility criteria, and then began to register those who were willing to take part in the study. Then more detailed information on the goals, importance, and advantages of participation in the study, as well as the research implementation stages, was provided. If they were willing to participate regularly and continuously in the study, the pre-pregnancy or the first trimester weight of pregnant women extracted from their health files and was recorded in the relevant checklist and the perceived personal resource questionnaire was completed by pregnant women. Then women with a poor and moderate perceived social support score (125 or less) and their spouses were asked to sign a consent form if they were willing to participate in the study. After completing the written and informed consent form by the pregnant women and their husbands, other questionnaires including demographic information, perceived stress, and Spielberger’s state and trait anxiety were completed by them.

Fathers who participated in the study were randomly assigned to two groups, namely counseling and control through blocking in four and six blocks using www.random.org website and allocation ratio of 1:1. Blocking was done by a person who was not involved in data sampling and analysis. To conceal the allocation, the type of intervention was written on a paper and placed in opaque envelopes.

### Intervention

Four 60-min sessions of group counseling were held in 7–10 member groups once a week for four consecutive weeks by the researcher in order to familiarize fathers with changes in pregnancy and their role in maternal and fetal health. The outlines of counseling sessions included:

#### First session

The effect of social support on mother and fetus during pregnancy, the role of fathers in supporting the pregnant mothers, the effects of not supporting pregnant women and its outcomes after birth, the importance of fathers’ role during pregnancy, the importance of fathers’ relationship with fetus and duties of fathers during pregnancy and childbirth, and sexual relations and their changes in pregnancy.

#### Second session

Mental health during pregnancy, anatomical, physiological, and hormonal changes during pregnancy, and the effect of such changes on the body, and especially the mother’s mental state, and providing solutions to better cope with these changes, fathers’ role in helping mothers to adapt to changes during pregnancy.

#### Third session

Familiarity with the stages of fetus development during pregnancy, the effect of pregnant mothers’ nutrition and fathers’ attention to the feeding of their pregnant wives during pregnancy, and the effect of prenatal care on maternal and fetal health.

#### Fourth session

Risk signs and symptoms during pregnancy and dealing with them, delivery process and stages, delivery methods (cesarean section and normal) and their advantages and disadvantages, and preparation of fathers and mothers for delivery.

About 70% of fathers attended regularly in counseling sessions and for fathers who for any reason were unable to attend some of the sessions, an audio file of the counseling sessions was provided to be able to use the content of counseling sessions. Also, the contact number of the consultant (first author) was provided to the participants to call if they have any questions. Four weeks after the intervention, the questionnaires were filled out again by the participants in the control and intervention groups.

### Data collection tools

The data collecting tools in this study were the demographic questionnaire, the Perceived Personal Resource Questionnaire-85-Part 2 (PRQ-85-Part 2), the Perceived Stress Scale (PSS) and the Spielberger’s State-Trait Anxiety Inventory (STAI).

The demographic questionnaire included questions about age, education, occupation, spouse’s occupation, gestational age, mother’s weight, and mother’s height.

In this study, the PRQ-85-Part 2 was used to assess social support. PRQ-85 was developed by Brandt & Weinert [24] and includes two parts. The second part includes 25 items that are scored based on the Likert scale from 1 (strongly disagree) to 7 (strongly agree). The range of scores is from 25 to 175.

The PSS was developed by Cohen et al. in 1983 [25] and has 3 versions of 4, 10, and 14 items that are used to measure the general perceived stress in the past month. PSS measures the thoughts and feelings about stressful events, control, overcoming, coping with stress, and experienced stresses. Each item has 5 options based on the Likert scale (never = 0, almost never = 1, sometimes = 2, fairly often = 3, very often = 4). In this study, the 14 items version was used. The total score ranges between zero and 56. It should be noted that 7 items in this scale (4, 5, 6, 7, 9, 10, 13) have a positive meaning and are scored inversely (never = 4, almost never = 3, sometimes = 2, fairly often = 1, very often = 0).

The STAI includes of 40 items in two parts. The first part measures the state anxiety and includes of 20 items scored with the 4-point Likert scale (never =1 to always = 4). The second part of STAI is used to measure trait anxiety and also consists of 20 items (21–40) scored with the 4-point Likert scale (almost never = 1 to almost always = 4). Scores of state and trait anxieties are calculated separately and summed [26]. The minimum and maximum scores for items 1 to 20 of state and trait forms are 20 and 80, respectively.

### Data analysis

The data was analyzed by using SPSS version 24. Normality of the quantitative data was assessed using Kolmogorov-Smirnov test and all data has normal distribution. The socio-demographic characteristics were compared through independent t, Chi-square, Chi-square for trend and Fisher exact tests. The independent t-test was used to compare the mean scores of stress and anxiety before intervention, and the ANCOVA with baseline score adjustment was to compare the mean scores of stress and anxiety 4 weeks after intervention. All tests were done based on intention-to-treat and *p* < 0.05 was considered significant.

## Results

**Fig. 1:**
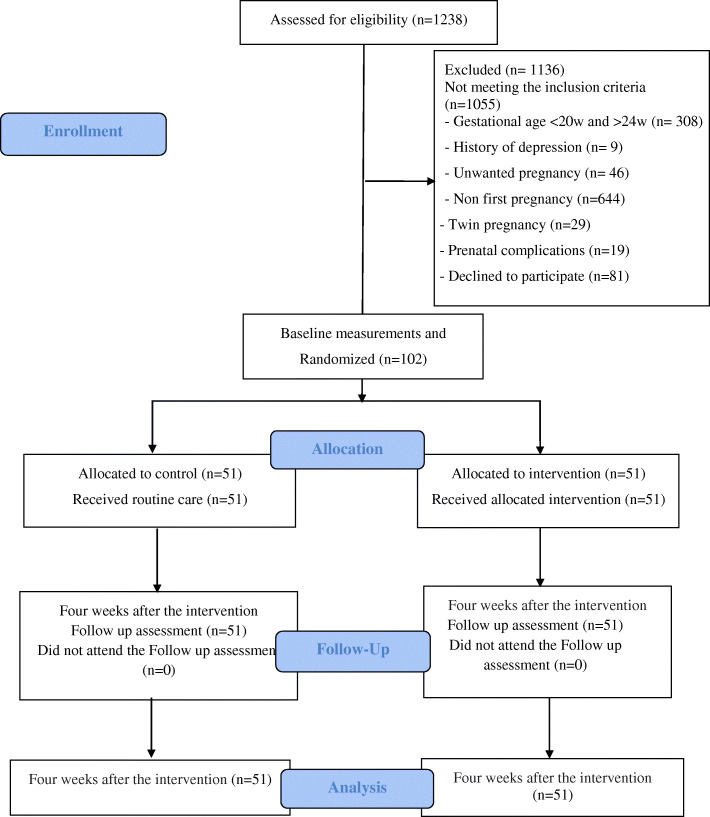
Flowchart of the study

Figure 1 shows the study flowchart. There was no significant difference between the two groups in terms of socio-demographic characteristics (*p* < 0.05). The mean age of the participants in the intervention and control groups was 33.2 ± 5.8 and 32.9 ± 6.2 years, respectively. One third of the participants in the intervention group (35.3%) and more than one-fifth of those in the control group (23.5%) had academic degrees. Only one participant (2%) in the intervention group was unemployed. In terms of income status, most of the participants in the intervention (80.4%) and control groups (70.6%) stated that they have sufficient income (Table 1).

**Table 1:** Socio-demographic characteristics of participants in counseling and control groups

Variable	Counseling (***n*** = 51) Number (percent)	Control (***n*** = 51) Number (percent)	***P***-value
**Age** (year)^a^	33.2 (5.8)	32.9 (6.2)	0.758^*^
**Education**			0.662^**§**^
Illiterate	1 (2.0)	1 (2.0)	
Primary school	6 (11.8)	3 (5.9)	
Secondary school	4 (7.8)	8 (15.7)	
High school	6 (11.8)	7 (13.7)	
Diploma	16 (31.4)	20 (39.2)	
University	18 (35.3)	12 (23.5)	
**Job**			0.710^‡^
Jobless	0 (0.0)	1 (2.0)	
Worker	12 (23.5)	17 (33.3)	
Employed	9 (17.6)	7 (13.7)	
Shopkeeper	7 (13.7)	6 (11.8)	
Other	23 (45.1)	20 (39.2)	
**Income sufficiency**			0.162^**§**^
Completely sufficient	8 (15.7)	7 (13.7)	
Almost sufficient	41 (80.4)	36 (70.6)	
Never sufficient	2 (3.9)	8 (15.7)	

^a^Numbers are reported in terms of mean (standard deviation)

*independent t-test ‡ Fisher’s exact test § Chi-square for trend test

Before intervention, there was no significant difference between the two groups in terms of perceived stress (*p* = 0.115), state anxiety (*p* = 0.817), and trait anxiety (*p* = 0.789). Based on the ANCOVA (with baseline score adjustments), the mean scores of state anxiety in the intervention group was significantly lower than that of the control group (Adjusted Mean Difference (AMD): -2.4; 95% Confidence Interval (95% CI): − 4.7 to − 0.2; *p* = 0.030), and there was no significant difference between the two groups in terms of trait anxiety (AMD: -0.8, 95%CI: − 3.2 to 1.5, *p* = 0.472) and perceived stress (AMD: -0.4, 95%CI: − 2.5 to 1.4, *p* = 0.635) (Table 2).

**Table 2:** Comparison of mean score of stress, state and trait anxiety before and 4 weeks after intervention in the studied groups

Variable	Counseling (*n* = 51) Mean (SD^a^)	Control (*n* = 51) Mean (SD^a^)	AMD (95% CI)^b^	*P*
**Perceived stress (0–56)**				
Before intervention	22.5 (7.8)	20.1 (7.4)	2.4 (−0.6 to 5.4)	0.115
Four weeks after the intervention	21,5 (9.3)	19.7 (8.6)	−0.4 (−2.5 to 1.5)	0.635
**State anxiety (20–80)**				
Before intervention	36.1 (10.3)	36.6 (10.9)	-0.4 (4.6 to 3.6)	0.817
Four weeks after the intervention	34.6 (10.0)	37.5 (12.2)	−2.4 (− 4.7 to −0.2)	0.030
**Trait anxiety (20–80)**				
Before intervention	36.1 (10.3)	35.6 (10.9)	0.5 (−3.6 to 4.7)	0.789
Four weeks after the intervention	35.9 (11.0)	36.2 (12.1)	−0.8 (− 3.2 to 15)	0.472

^a^Standard Deviation, ^b^Adjusted Mean Difference (95% Confidence Interval)

Independent t-test was used to compare the groups before intervention. After intervention, ANCOVA test with baseline control was used

## Discussion

Based on the findings, holding counseling sessions with fathers can help expectant fathers to manage their feelings, meet their needs, and reduce their anxiety during pregnancy.

Charandabi et al. (2017) in their clinical trial entitled “the effect of lifestyle-based education on fathers’ anxiety and depression during pregnancy and postpartum periods,” [27] found that the mean score of anxiety in the intervention group was significantly lower than that of the control group, which is consistent with the results of the present study. This result confirms the fact that pregnancy is a challenging period not only for mothers, but also for expectant fathers [28]. That is accompanied with fear and concerns about the health of mothers and babies [17]; however, prenatal clinics mostly focus on maternal and infant health, and often ignore the needs and feelings of expectant fathers [19]. This is while, at the beginning of the pregnancy period, fathers need to attend more counseling sessions [9] to reduce their stress and anxiety.

Other similar studies have been conducted on the effect of preventive measures on mental health, especially during pre/postpartum periods. For example, it was found in a study that physical activity can effectively prevent anxiety [29]. In another study, physical activity was associated with improved mental health, reduced anxiety scores, and increased self-esteem [30]. The results of another study indicated that education and exercise can effectively improve physical and mental health and reduce anxiety [31]. In another study, researchers found that education can reduce anxiety through increasing participants’ knowledge and changing their attitude and behavior [32]. These results are consistent with the present results and indicate the positive role of education and support during pre/postpartum periods; therefore, it seems that more attention should be paid to emotional problems of fathers in order to prevent their mental and emotional problems during pregnancy and childbirth, and finally help men and women successfully adapt to their new parenting roles.

The present results showed that counseling to fathers does not affect their stress during pregnancy. Unfortunately, few interventional studies have been carried out on men during the pregnancy of their spouses [26, 33, 34]. The results of some descriptive studies are presented below, which indicate high levels of stress in men during this period. This highlights the importance of appropriate educational and counseling interventions for men during pregnancy.

The results of a study conducted on 300 pregnant women and their spouses showed that men, like their wives, display signs of stress and anxiety during pregnancy. In this study, higher levels of stress and anxiety were observed especially during the first 3 months [35]. The results of a longitudinal study showed high levels of stress during pregnancy, especially among fathers who had attended a few childbirth classes. The results of this study showed that fathers with higher levels of fear had higher levels of stress, as well as poorer physical and mental health conditions [36]. In another study conducted on 312 expectant fathers, higher levels of psychological distress were observed in men during pregnancy. Fathers who have insufficient information about pregnancy and childbirth are subject to more distress, and poor marital relationships and social support are associated with psychological distress [15]. In a longitudinal study, 221 men whose pregnant wives were admitted to a major obstetric hospital in Melbourne were selected, and completed the anxiety, stress, and social support questionnaires in mid-pregnancy, late pregnancy, and postpartum periods. Based on the results, the expectant fathers experienced high levels of stress and anxiety during pregnancy and lower levels after childbirth [13]. The above results indicate that fathers, like mothers, suffer from stress during pregnancy, and since fathers’ health affects the overall family health, specialists are recommended to address their emotional problems during this period.

Since the low social support during pregnancy is due to the lack of awareness and non-participation of fathers in the pregnancy process and programs [15, 37], in the present study, fathers whose pregnant wives received moderate and low social support scores were selected as the target population. Therefore, it is recommended that such fathers and their pregnant wives be considered more when providing counseling and educational programs during pregnancy. In the present study, group counseling method was used, and the content of the counseling sessions was about physical and mental changes in pregnancy, fetal development and prenatal care. Group counseling is a form of counseling that group members can learn from the experiences of others and share their experiences with them, and can experience new behaviors through each other’s emotional support [38]. Also, based on some studies, lack of awareness about the process of pregnancy, fetal growth and development, signs and symptoms of pregnancy and prenatal care are the causes of stress and anxiety of fathers [15, 37]. Therefore, it seems that increasing the awareness and participation of fathers in the pregnancy process can be effective in reducing fathers’ anxiety.

At present, most of Iranian men have traditional marriages. They are the head of a family in Iranian culture and have special respects and having children is important for most of them [39]. Although, gender differences are still prominent among Iranian men [40], however, the effect of globalization is evident in the daily life of Iranian women [41]. With Iranian women entrance into the work field, the role of fathers related to their children has become more important [39]. In Iran, similar to other countries, the participation of fathers during the perinatal period is encouraged [42]. In a study in Iran, fathers identified themselves responsible for health and supplying the child’s needs. They believed in participation in childrearing. Therefore, educational programs are recommended to help fathers in accepting and performing their responsibilities [43].

In this study, most of the fathers were employed and a major portion of them had high school diplomas and academic degrees; this may affect the generalizability of the results to illiterate and unemployed men. Some strengths of this study included using random selection and allocation method and allocation concealment technique, selecting the participants among all health centers across the study area, using the participants’ native language during counseling sessions, providing the participants with a telephone number and answering their questions.

## Conclusion

In general, the results indicate that counseling reduced state anxiety of expectant fathers. Therefore, counseling techniques such as educational and supportive methods can be used to increase the awareness of fathers about pregnancy and childbirth and help expectant fathers manage their feelings, meet their needs, and reduce their anxiety during pregnancy in order to prevent adverse pregnancy and childbirth outcomes. In addition, maternity-care policy makers must pay more attention to the feelings and concerns of fathers during pregnancy and hold counseling sessions for them and invite them to participate in pregnancy and childbirth care procedures. They should also develop programs to raise the awareness of health care providers about the important role of fathers in preventing relevant adverse outcomes. Adding counseling sessions for expectant fathers to prenatal care programs can effectively improve the overall health of mothers, fathers, and infants.

## Data Availability

The datasets used and/or analyzed during the current study are available from the corresponding author on reasonable request.

## Abbreviations

AMD: Adjusted Mean Difference
MD: Mean Difference
PRQ: Personal Resource Questionnaire
STAI: State-Trait Anxiety Inventory
PSS: Perceived Stress Scale
